# Step by step: Association of device‐measured daily steps with all‐cause mortality—A prospective cohort Study

**DOI:** 10.1111/sms.13726

**Published:** 2020-06-17

**Authors:** Bjørge Herman Hansen, Knut Eirik Dalene, Ulf Ekelund, Morten Wang Fagerland, Elin Kolle, Jostein Steene‐Johannessen, Jakob Tarp, Sigmund Alfred Anderssen

**Affiliations:** ^1^ Department of Sports Medicine The Norwegian School of Sport Sciences Oslo Norway

**Keywords:** all‐cause mortality, device‐measured, prospective cohort, steps, walking

## Abstract

**Introduction:**

Walking is free, does not require special training, and can be done almost everywhere. Therefore, walking is a feasible behavior on which to tailor public health messages. This study assesses the prospective association and dose‐response relationship between daily steps and all‐cause mortality.

**Materials and Methods:**

Daily steps were measured by waist‐mounted accelerometers in 2183 individuals (53% women) for seven consecutive days at baseline (2008‐09). Participants were followed for a median period of 9.1 years and associations between steps and all‐cause mortality determined by registry linkage were assessed using Cox proportional hazard regression with adjustment for relevant covariates.

**Results:**

Mean age was 57.0 (SD 10.9) years at baseline. Median (IQR) daily steps across ascending quartiles were 4651 (3495‐5325), 6862 (6388‐7350), 8670 (8215‐9186), and 11 467 (10 556‐13 110), respectively. During follow‐up, 119 individuals died (68% men). Higher number of daily steps was associated with a lower risk of all‐cause mortality with hazard ratios of 1.00 (referent), 0.52 (0.29‐0.93), 0.50 (0.27‐0.94), and 0.43 (0.21‐0.88) across ascending quartiles of daily steps in the multivariable‐adjusted model with follow‐up commencing 2 years after baseline. Risk differences per 1000 individuals for ascending quartiles were 6.8 (2.9‐9.3), 7.1 (0.8‐11.1), and 8.0 (1.7‐12.1), respectively.

**Conclusions:**

Daily steps were associated with lower mortality risk in a non‐linear dose‐response pattern. The risk is almost halved when comparing the least active referent against the second quartile equivalent to a difference of about 2200 daily steps. Encouraging those least active to increase their daily steps may have substantial public health implications.

## 1 INTRODUCTION

Walking—a basic unit of locomotion—is free, does not require special training, and can be done almost everywhere.^1^ Furthermore, number of steps taken also has the advantage that it can be selfmonitored by most smartphones or other wearables, making a feasible behavior on which to tailor public health messages. Walking has been associated with lower incidence of all‐cause mortality^2^, ^3^ and cardiometabolic risk,^4^ but most studies addressing the longevity benefits associated with walking are limited by the use of self‐reported indicators of steps taken per day, time spent walking per day^5^, ^6^ or have not quantified the dose‐response relationship between steps taken and subsequent reduction in risk of all‐cause mortality—with some notable exceptions. Dwyer et al^7^ showed a linear decrease in risk for all‐cause mortality with more accumulated daily steps after 10 years of follow‐up. Yamamoto et al^8^ showed that older adults in the highest quartile of daily steps had a lower risk of death compared to the least active quartile, with no linear relationship between steps and all‐cause mortality were observed, the latter potentially attributable to a modest sample size. Lee at al^9^ showed marked risk reductions for all‐cause mortality associated with as few as 4400 daily steps compared to the referent after 4 years of follow‐up in a sample of older US women. Similar results were recently reported in a nationally representative sample of US adults (40 years and older).^10^ However, most previous studies included mainly older individuals and were non‐nationally representative samples.^8^, ^9^, ^11^ Thus, additional studies including nationally representative samples including potentially more active middle‐aged and older individuals are warranted.

The US surgeon general recently announced a call to action focused on walking and the walkability of communities for overall health in an effort to increase moderate and vigorous physical activity,^12^ and the recent US physical activity guideline advisory committee asked for more research on the association between steps and health.^13^ Identifying factors associated with healthy aging that can be translated into intuitive and absolute metric‐based recommendations is of vital importance to both policymakers as well as public health workers. Therefore, the aim of the present study was to assess the prospective dose‐response relationship between device‐measured daily steps and all‐cause mortality in a population‐based cohort of middle‐aged and older adults.

## 2 MATERIALS AND METHODS

### 2.1 Data source and study population

A detailed description on study population, sampling, and methods is found elsewhere.^14^ Briefly, in 2008 and 2009, we conducted a nationwide multicenter physical activity surveillance study involving 10 regional test centers throughout Norway. A representative sample of 11 515 adults and older people (20‐85 years) was drawn from the Norwegian population registry. The study information and informed consent were distributed via mail to the sample; 267 invitations were returned because of an unknown address. This resulted in an eligible sample of 11 248 individuals invited to participate, and written informed consent was obtained from 3867 individuals (34%). Three hundred eighty‐two subjects did not return any data, giving a final sample of 3485 individuals (31%). The study was approved by the Regional Ethics Committee for Medical Research and the Norwegian Social Science Data Services. We conducted the study according to the Helsinki declaration. For the present analyses, we restricted the sample to individuals aged 40 or older (n = 2475) with valid data across exposure variables and covariates (n = 2183).

### 2.2 Anthropometry and demographics

Participants self‐reported their sex, height, and weight (to nearest millimeter and 0.1 kg, respectively). We calculated body mass index (BMI) as body weight (kg)/height (m^2^), and classified participants according to the WHO classification.^15^ We used self‐reported education level as a proxy for socioeconomic status and collapsed a six‐category item into three groups: low (primary school, lower secondary school, vocational high school), middle (secondary school/high school), or high (undergraduate or graduate degree). Furthermore, self‐reported alcohol consumption status (frequency; never, monthly or rarer, 2‐4 times per month, 2‐3 times per week, 4 times per week or more), self‐reported smoking status (never, former or current), and self‐reported history of medical conditions (continuous sum derived from the item “Have your doctor diagnosed you with (any of the following): diabetes type 1, diabetes type 2, congestive heart failure, coronary heart disease, angina/angina pectoris, heart attack, stroke, cancer or malignancy”).

### 2.3 Daily steps

The ActiGraph GT1M physical activity monitor (ActiGraph, LLC) was used to assess the participants’ number of daily steps taken and intensity‐specific physical activity. Participants were instructed to wear the device for seven consecutive days while awake, except during water‐based activities (eg, showering and swimming). The accelerometers were initialized and downloaded using the ActiLife software provided by the manufacturer (ActiGraph, LLC), and data were collected at 30 Hz using the normal filter in 10‐s epochs. All data were reintegrated into 60‐seconds epochs using a specialized accelerometer analytical software (Kinesoft, version 3.3.80). Non‐wear time was defined as intervals of at least 60 consecutive minutes with zero counts, with allowance for 1‐2 minutes with activity counts above 0. Daily steps were determined using the manufacturer`s step algorithm and have demonstrated acceptable validity when compared to a criterion method.^16^ Furthermore, vigorous physical activity (VPA) was defined as all recorded activity at ≥5999 counts per minute.^17^ Individuals were included in the analysis if they recorded at least 4 days of at least 10 hours of accelerometer data per day.

### 2.4 Mortality

Participants were followed for all‐cause mortality with a median follow‐up of 9.1 years. Follow‐up time was counted from the first day of valid accelerometry data (2008‐2009) up over a median period of 9.1 years with all‐cause mortality ascertained from the Norwegian Cause of Death Registry which cover about 98% of all deaths in Norway.^18^ To reduce the impact of possible reverse causation bias, we also included a model with follow‐up starting 2 years after the first day of valid accelerometry data.

### 2.5 Statistics

We used cox proportional hazards regression models to estimate hazard ratios (HR) and 95% confidence intervals (CI`s) for the associations between daily steps in quartiles (using the least active quartile as reference) and all‐cause mortality with age as the time scale.^19^ The models were adjusted for the following covariates: model A: sex, and minutes of valid wear time per day; model B: model A + time (min/day) spent in VPA (VPA was modeled as a covariate to take more vigorous intensity activity (eg, jogging) into account); model C: model B + level of education and BMI (continuous); model D: model C + alcohol consumption, smoking status, and number of medical conditions. In our final model E (adjusted as model D), follow‐up was commenced 2 years after baseline. A test of trend was performed by assigning the quartile‐median value to all participants in the quartile and modeling these as a continuous variable. We noticed no violation of the proportional hazards assumption in visual inspection of log‐log plots and Schoenfeld residuals plotted against follow‐up time. The dose‐response relationship between daily steps and all‐cause mortality was assessed using a restricted cubic spline model to allow for potential non‐linearity, with prespecified knots placed at the 10th, 50th, and 90th centiles of the step distribution. Departure from linearity was assessed by a Wald test examining the null hypotheses that the coefficient of the second spline was equal to zero. We calculated adjusted absolute risk differences from the crude baseline risk and the adjusted risk ratio.^20^ All analyses were performed using Stata 13.1 (StataCorp. 2013. Stata Statistical Software: StataCorp LP.). Values of p were two‐sided with a significance level of 0.05.

## 3 RESULTS

After removal of 141 individuals with less than 4 days of valid physical activity measurements and 151 individuals with one or more missing covariate, the analytical sample consisted of 2183 individuals. The mean age of the sample was 57.0 years (SD 10.9), with 43.2% of women and 61.9% of men categorized as either overweight or obese. The baseline characteristics of participants across quartiles of daily steps are displayed in table 1. In brief, age, BMI, and prevalence of self‐reported medical conditions differed across quartiles. Median (IQR) steps taken per day among the 25% least active (Q1) were 4651 (3495, 5325), whereas the most active quartile accumulated a median of 11 467 (10 556, 13 110) steps per day.

**TABLE 1:** Baseline characteristics of participants by quartiles of daily steps

	Quartiles of daily steps (range)				Total
	Quartile 1 (≤5922)	Quartile 2 (5922‐7743)	Quartile 3 (7744‐9842)	Quartile 4 (≥9843)	
No. of participants	545	546	546	546	2183
Daily steps; median (IQR), mean (SD)	4651 (3495, 5325)	6862 (6388, 7350)	8670 (8215, 9186)	11 467 (10 556, 13 110)	8002 (3113)
Wear days; mean (SD)	6.8 (1.0)*	7.0 (0.9)	6.8 (0.7)	6.9 (0.8)	6.9 (0.8)
Wear minutes/day; mean (SD)	840 (70)**	878 (56)	891 (54)	900 (54)	877 (63)
Age (years); mean (SD)	62.5 (12.3)***	55.7 (10.4)	54.8 (9.8)	54.9 (8.9)	57.0 (10.9)
Sex					
Women	283 (52)	268 (49)	296 (54)	310 (57)	1157 (53)
Men	262 (48)	278 (51)	250 (46)	236 (43)	1026 (47)
BMI; mean (SD)	26.7 (4.7)^†^	26.3 (3.5)	25.3 (3.5)	24.5 (3.3)	25.7 (3.9)
BMI category^a^; n (%)^a^					
Underweight	5 (0.9)	1 (0.2)	0	11 (2)	17 (0.8)
Normal weight	208 (38)	216 (40)	289 (53)	318 (58)	1031 (47)
Overweight	225 (41)	251 (46)	211 (39)	181 (33)	868 (40)
Obese	107 (20)	78 (14)	46 (8)	36 (7)	267 (12)
Level of education^b^; n (%)^b^					
Low	151 (28)	86 (16)	78 (14)	69 (13)	384 (18)
Middle	207 (38)	216 (40)	195 (36)	219 (40)	837 (38)
High	187 (34)	244 (45)	273 (50)	258 (47)	962 (44)
Smoking status; n (%)^b^					
Current	135 (25)	99 (18)	79 (14)	81 (15)	394 (18)
Former	203 (37)	227 (42)	201 (37)	179 (33)	810 (37)
Never	207 (38)	220 (40)	266 (49)	286 (52)	979 (45)
Alcohol consumption; n (%)^b^					
Never	70 (13)	36 (7)	35 (6)	43 (8)	184 (8)
Monthly or rarer	158 (29)	122 (22)	108 (20)	106 (19)	494 (23)
2‐4 times per month	181 (33)	224 (41)	212 (39)	211 (39)	828 (38)
2‐3 times per week	106 (19)	128 (23)	163 (30)	145 (27)	542 (25)
4 times per week or more	30 (6)	36 (7)	28 (5)	41 (7)	135 (6)
Self‐reported illnesses; n (%)					
Diabetes type 1^†††^	11 (2)	4 (0.7)	3 (0.6)	2 (0.4)	20 (0.9)
Diabetes type 2^††^	49 (9)	16 (3)	10 (2)	14 (3)	89 (4)
Cancer^†††^	25 (5)	11 (2)	14 (3)	7 (1)	57 (3)
CVD^c^, ^††^	113 (21)	56 (10)	39 (7)	41 (8)	249 (11)

^a^ According to WHO classification.^15^

^b^ Low: primary school, lower secondary school, vocational high school; middle: secondary school/high school; high: undergraduate or graduate degree.

^c^ Congestive heart failure, coronary heart disease, angina/angina pectoris, heart attack, or stroke.

* *P* < .01 compared to Q2.

** *P* < .01 between all step quartiles.

*** *P* < .001 compared to Q2‐4.

^†^ *P* < .001 compared to Q3‐4.

^††^ *P* < .001 (Pearson chi‐square).

^†††^ *P* < .05 (Pearson chi‐square).

During a median follow‐up of 9.1 years, 119 individuals died (68% men). Adjusted for age and sex, the HRs (95% CI) for increasing quartiles of daily steps were 1.00 (reference), 0.42 (0.24‐0.74), 0.47 (0.26‐0.84), and 0.40 (0.21‐0.76), respectively (*P* for trend = 0.02) (table 2). Adjustment for VPA did not alter any associations (model B), and results were robust to increasing levels of adjustment with HRs across quartiles for the multivariable‐adjusted model (model D) of 1.00 (ref), 0.45 (0.26‐0.81), 0.49 (0.27‐0.89), and 0.42 (0.21‐0.84), respectively (*P* for trend = 0.04). Our final model (model E) with follow‐up commencing 2 years after baseline yielded only slightly attenuated HRs of 1.00 (reference), 0.52 (0.29‐0.93), 0.50 (0.27‐0.94), and 0.43 (0.21‐0.88) (*P* for trend = 0.01), indicating a risk reduction of 48% for all‐cause mortality between the least active individuals (median steps and IQR: 4651 [3495, 5325]) and the second least active individuals (6862 [6388, 7350]). Risk differences per 1000 individuals (95% CI) between quartile 1 and quartiles 2‐4 were 6.8 (2.9‐9.3), 7.1 (0.8‐11.1), and 8.0 (1.7‐12.1), respectively.

**TABLE 2:** Hazard ratios (95% CIs) for all‐cause mortality by quartiles of daily steps (range)

	Quartile 1	Quartile 2	Quartile 3	Quartile 4	*P* for trend
Daily steps (range^a^)	<6000	6000‐<8000	8000‐<10 000	≥10 000	
No. of participants (cases)	545 (73)	546 (17)	546 (16)	546 (13)	
Model A^b^	Ref	0.42 (0.24‐0.74)	0.47 (0.26‐0.84)	0.40 (0.21‐0.76)	<.01
Model B^c^	Ref	0.43 (0.24‐0.74)	0.48 (0.27‐0.86)	0.43 (0.22‐0.83)	<.01
Model C^d^	Ref	0.41 (0.23‐0.72)	0.45 (0.25‐0.82)	0.38 (0.20‐0.75)	<.01
Model D^e^	Ref	0.45 (0.26‐0.81)	0.49 (0.27‐0.89)	0.42 (0.21‐0.84)	<.01
No. of Participants (cases)	543 (66)	544 (17)	543 (15)	544 (12)	
Model E^f^	Ref	0.52 (0.29‐0.93)	0.50 (0.27‐0.94)	0.43 (0.21‐0.88)	.01

^a^ Range is rounded to the nearest 500 for communicative purposes, see Table 1 for exact range.

^b^ Sex and wear time.

^c^ Sex, wear time and VPA.

^d^ Sex, wear time, VPA, education and body mass index.

^e^ Sex, wear time, VPA, education, body mass index, smoking (never/former/current), alcohol intake, and number of medical conditions.

^f^ Sex, wear time, VPA, education, body mass index, smoking (never/former/current), alcohol intake, and number of medical conditions, excluding deaths within first 2 y (n = 9).

**FIGURE 1:**
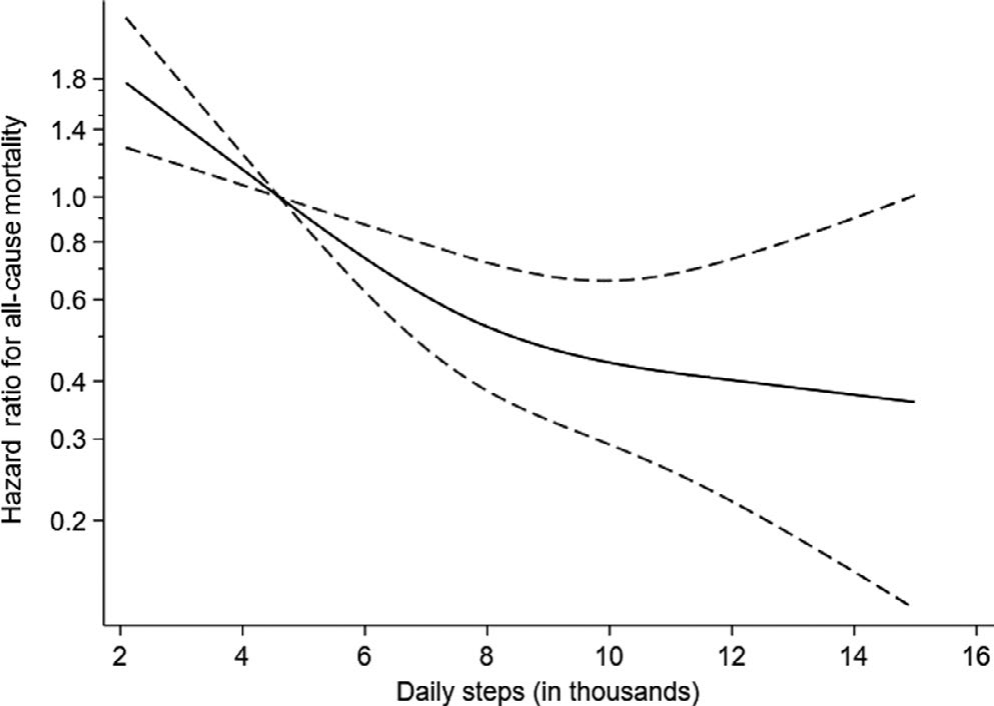
Dose‐response relationship between daily steps and all‐cause mortality (hazard ratios (solid line) with 95% CIs (dotted lines)). The y‐axis is a log scale and the x‐axis is truncated at 15 000 daily steps. Reference is median daily steps of the least active quartile

Figure 1 displays the dose‐response relationship between daily steps and all‐cause mortality modeled in continuous form, using 4600 steps per day as reference (≈median step count in quartile 1). We observed a non‐linear, dose‐response association between daily steps and mortality (*P*‐value from the second spline <0.001). The mortality risk was markedly reduced up to about 8000‐9000 daily steps, and higher levels only marginally reduced the risk further throughout the steps per day spectrum examined although no apparent plateauing of the relationship was evident within the observed variation in the exposure.

## 4 DISCUSSION

In this study of a national population‐based sample of women and men aged 40‐85 years at enrollment, we observed a non‐linear dose‐response relationship between daily steps and all‐cause mortality. The risk of all‐cause mortality was 48% lower for a difference of approximately 2200 daily steps in the second least active quartile compared with the least active referent, after taking major putative confounding factors into account and excluding the first 2 years of follow‐up to minimize reverse causation bias.

The present study confirms and extends current knowledge by including a large, population‐based longitudinal sample consisting of predominantly healthy women and men in which device‐measured daily steps were available. Few previous studies have assessed the association between device‐measured daily steps and mortality. Yamamoto et al^8^ reports a reduced risk of 54% for all‐cause mortality when comparing the least and most active pedometer‐determined quartiles (3394 vs 10 241 steps per day) in a sample of 421 older Japanese individuals (mean age 71 years). However, no dose‐response relationship between walking and mortality was observed, likely attributable to a small sample size and selection bias as stated by the authors. Dwyer at al^7^ assessed steps for two days using a pedometer in 2576 individuals (mean age 59 at inclusion). After a follow‐up period of 10 years, they observed an inverse and linear dose‐response relationship between daily steps and all‐cause mortality, showing a risk reduction of 6% per 1000 steps per day increase. Lee et al^9^ showed similar associations between accelerometer‐assessed daily steps and all‐cause mortality as Dwyer et al, but of greater magnitude, after 4.3 years of follow‐up in a sample of more than 16 000 older women (mean age of 71 years).^9^ With median daily steps across low to high quartiles of 2718, 4363, 5905, and 8842, respectively, they observed a 49% risk reduction between quartile 1 and quartile 2 and a dose‐dependent relationship between walking and mortality that levelled off at approximately 7500 daily steps. The similarities between the study by Lee et al^9^ and the present study indicate that the associations extend to younger individuals as well as men, albeit the absolute activity levels needed to elicit the same benefits are higher in our sample, likely explained by differences in age and activity levels. Lastly, the risk reductions observed in the present study are similar to those recently reported by Saint‐Maurice et al^10^ using a sample from the 2003‐2006 cycle of the National Health and Nutrition Examination Survey. Among US adults, individuals taking 8000 steps per day had significantly lower risk for all‐cause mortality, compared to the referent group taking 4000 steps per day (HR, 0.49 [95% CI, 0.44‐0.55]). Taken together, these results strongly indicate a substantially lower risk for all‐cause mortality associated with number of steps per day.

A major strength of the present study is the population‐based sample of adult and older participants, and the use of device‐measured steps over at least 4 days, including adjustment for vigorous physical activity. Because of known difficulties with accurately recalling details about physical activity and sedentary time,^21^ device‐based measurements are considered a preferable option in large‐scale studies.^22^ This is highlighted by findings of associations greater in magnitude reported here and elsewhere^23^ compared to what has been shown in studies that have used self‐reported indices of physical activity.^24^ This is likely due to regression dilution bias as a result of imprecision in exposure assessment.^25^ Furthermore, the temporal relationship between exposure (daily steps) and outcome (dead/alive) is clear and therefore the study design, albeit being observational by nature and assuming no unmeasured or residual confounding, selection bias or information bias, can be used to suggest causality. Nevertheless, it is not possible to rule out reverse causation (eg, that number of daily steps might be low due to present illness or poor health). In order to address this, we adjusted for relevant prevalent chronic conditions at baseline and started follow‐up 2 years after baseline in the final model. HRs were materially unchanged suggesting findings are not attributable to reverse causation bias. Lastly, it should be acknowledged that steps measured by the device may be due to physical activities other than walking and can be accumulated during a range of activities (eg, gardening, household chores, sports, dancing, active play).

Limitations include self‐reported covariates, relatively few cases, and the observational nature of the study. The results are likely affected by unmeasured and residual confounding and other biases such as reverse causation bias from a higher number of daily steps reflecting better health. Results were robust to the exclusion of deaths within the first 2 years but we were unable to adjust for mobility limitations and the severity of chronic conditions (data not available) which might have inflated our estimates. Furthermore, we acknowledge a relatively low response rate in the included sample. Among adults and older people, 31% of the invited sample participated, and the included sample differed compared with the underlying population according to socio‐demographic variables (income and level of education).^14^ This is common in population‐based surveys,^26^, ^27^ and we cannot rule out the possibility that daily steps in the included sample are somewhat higher than in the general population because of selection bias but this will not necessarily result in biased effect estimates.^28^ It should also be mentioned that the study only includes a single assessment of accelerometer‐determined steps, and we are not able to investigate stability of stepping behavior over time or impact of altering this behavior on risk of dying prematurely. Albeit being an obvious limitation, studies have shown that individuals to a large extent maintain their relative ranks within the population over time.^8^, ^9^ Furthermore, accelerometers have been shown to underestimate daily steps in frail older individuals,^29^ potentially understating the amount of physical activity most beneficial for health in these populations.^30^ Lastly, we only investigated associations between daily steps and all‐cause mortality. More work is needed to determine the exact dose‐response relationships between steps and other chronic morbidities such as type 2 diabetes.

## 5 PERSPECTIVES

We observed a 48% lower risk for all‐cause mortality when comparing the least active individuals (referent) with the second least active quartile, with an absolute difference of 2200 daily steps between the two groups. Furthermore, we observed a non‐linear dose‐response association between daily steps and all‐cause mortality.

The present study extents current knowledge by confirming a clear and non‐linear dose‐response relationship between daily steps and mortality in a predominantly healthy sample of middle‐aged women and men with relative high activity levels compared to other samples.^31^ The benefits associated with a greater number of daily steps suggest creating walkable societies should be a key component of large‐scale primordial prevention efforts in the general population and the growing evidence‐base of the associations between daily steps and health has the potential for informing PA guidelines and underpins the importance of focusing public health efforts toward those most inactive. For example, 2200 steps (equivalent to the difference between the referent and the second quartile) translate to walking for an additional 1.6 km per day assuming a step length of 76 cm (2.5 ft). If confirmed, the substantial health gain observed, associated with moderate effort, may serve as encouragement to many sedentary individuals. Our findings underscore the potential of daily walking for longevity and reducing the economic burden of physical inactivity.

## CONFLICT OF INTERESTS

The authors declare that they have no competing interests.
